# Motor skills mediated through cerebellothalamic tracts projecting to the central lateral nucleus

**DOI:** 10.1186/s13041-019-0431-x

**Published:** 2019-02-08

**Authors:** Nobuyuki Sakayori, Shigeki Kato, Masateru Sugawara, Susumu Setogawa, Hotaka Fukushima, Rie Ishikawa, Satoshi Kida, Kazuto Kobayashi

**Affiliations:** 1 Department of Molecular Genetics, Institute of Biomedical Sciences, Fukushima Medical University, Fukushima, 960-1295 Japan; 2 AMED-CREST, Japan Agency for Medical Research and Development, Tokyo, 100-0004 Japan; 3 Department of Bioscience, Faculty of Life Science, Tokyo University of Agriculture, Tokyo, 156-8502 Japan

**Keywords:** Cerebellum, Dentate nucleus, Thalamus, Central lateral nucleus, Motor skill, Spatial recognition

## Abstract

The cerebellum regulates complex animal behaviors, such as motor control and spatial recognition, through communication with many other brain regions. The major targets of the cerebellar projections are the thalamic regions including the ventroanterior nucleus (VA) and ventrolateral nucleus (VL). Another thalamic target is the central lateral nucleus (CL), which receives the innervations mainly from the dentate nucleus (DN) in the cerebellum. Although previous electrophysiological studies suggest the role of the CL as the relay of cerebellar functions, the kinds of behavioral functions mediated by cerebellothalamic tracts projecting to the CL remain unknown. Here, we used immunotoxin (IT) targeting technology combined with a neuron-specific retrograde labeling technique, and selectively eliminated the cerebellothalamic tracts of mice. We confirmed that the number of neurons in the DN was selectively decreased by the IT treatment. These IT-treated mice showed normal overground locomotion with no ataxic behavior. However, elimination of these neurons impaired motor coordination in the rotarod test and forelimb movement in the reaching test. These mice showed intact acquisition and flexible change of spatial information processing in the place discrimination, Morris water maze, and T-maze tests. Although the tract labeling indicated the existence of axonal collaterals of the DN-CL pathway to the rostral part of the VA/VL complex, excitatory lesion of the rostral VA/VL did not show any significant alterations in motor coordination or forelimb reaching, suggesting no requirement of axonal branches connecting to the VL/VA complex for motor skill function. Taken together, our data highlight that the cerebellothalamic tracts projecting to the CL play a key role in the control of motor skills, including motor coordination and forelimb reaching, but not spatial recognition and its flexibility.

## Introduction

The cerebellum is a highly organized brain structure that contains a diverse range of neurons, which are involved in motor control and higher cognitive functions [1–4]. Purkinje cells are unique projection neurons in the cerebellum, and innervate the deep cerebellar nuclei, the sole cerebellar output channels. These cells in the lateral cerebellum mainly project to the dentate nucleus (DN), one of the deep cerebellar nuclei. Lesions of the lateral cerebellum or the DN disrupt visually guided locomotion in the obstacle avoidance test [5], spatial discrimination learning in the active avoidance test [6], and spatial cue learning in the Morris water maze test [7, 8], but not overground locomotion [5] or muscle strength [7]. Thus, the lateral cerebellum and the DN regulate complex motor control, being likely involved in spatial information processing.

The cerebellum processes neuronal information cooperating with other brain regions. Neurons in the DN innervate several thalamic nuclei, including the ventroanterior nucleus (VA) and ventrolateral nucleus (VL), which are the main motor thalamic areas having a vast amount of output to the motor cortex [9]. The DN neurons also innervate the central lateral nucleus (CL), one of the intralaminar thalamic nuclei [10–12]. The CL provides the projections to many brain regions, especially the motor cortex and dorsal striatum (DS) [13–16]. Recent studies indicate the role of the CL as a relay to connect the cerebellum and the DS; the DN modulates bi-synaptically the activity of the DS through the CL [17–19]. While these studies suggest the functional importance of the connection between the cerebellum and thalamus, it is still uncertain as to what kinds of behavioral components are processed through the pathway originating from the DN and projecting to the CL.

Here, we focused on the cerebellothalamic projection from the DN to the CL in mice, and evaluated its role in cerebellum-related behavioral tasks, including motor skills and spatial cue-related learning. We selectively eliminated the cerebellothalamic tracts using the immunotoxin (IT) targeting technology [20]. Here, we injected a lentiviral vector for neuron-specific retrograde gene transfer (NeuRet) [21], which carried the gene cassette encoding a human interleukin-2 receptor α-subunit (IL-2Rα), a receptor for a recombinant IT, into the CL, and subsequently injected the IT solution into the DN. The elimination of the cerebellothalamic tracts results in impaired motor coordination and forelimb reaching without affecting spontaneous locomotor activity or the gait pattern. We also found intact spatial recognition and its flexibility in two-lever place discrimination, Morris water maze, and T-maze tests. We further found the collateral axons to the VA/VL from the cerebellothalamic pathway, which had no detectable behavioral contribution. Collectively, these results demonstrate the important roles of the DN-CL tracts mainly in the control of motor skills, including motor coordination and forelimb reaching, forming the functional relay that connects the cerebellum and cortico-basal ganglia circuit.

## Methods

### Animals

C57BL/6J mice were obtained from Clea Japan (Tokyo, Japan), and were housed at Fukushima Medical University School of Medicine under a standard 12-h light/12-h dark schedule (lights on at 7:00 a.m.). Food and water were available ad libitum unless otherwise stated. All animal experiments were performed in accordance with the National Institutes of Health Guidelines for the Care and Use of Laboratory Animals, and were approved by our university’s committee for animal experimentation.

### Viral vector production

Culture of HEK293T cells, DNA transfection, and NeuRet lentiviral vector preparation were performed as described previously [21]. The NeuRet lentiviral vector was established using pseudo-typing with fusion glycoprotein type E [21]. The NeuRet lentiviral vectors carrying the gene cassette encoding the IL-2Rα fused to enhanced green fluorescent protein (EGFP) (NeuRet-IL-2Rα-EGFP) [22] and the cre recombinase with nuclear localization signal (NeuRet-Cre) [23] were used in this study. Preparation of adeno-associated viral vector serotype 2 (AAV2 vector) was performed as described previously [24]. The AAV2 vector containing a double-inverted *loxP*-flanked EGFP construct (AAV2-FLEX-EGFP) [25] was also used in this study.

For the titration of the NeuRet lentiviral vectors, viral RNA was isolated using a NucleoSpin RNA virus kit (Clontech, Mountain View, CA), and the copy number of the RNA genome was determined using a Lenti-X qRT-PCR titration kit and a StepOne real-time PCR system (Applied Biosystems, Tokyo, Japan) as described previously [21]. For the titration of the AAV2 vector, the viral genome titer was determined by quantitative PCR as described previously [24].

### Intracranial surgery

Mice were deeply anesthetized using isoflurane (1–2%), and each injection material was infused into the following brain regions through a glass microinjection capillary connected to a microinfusion pump (ESP-32; Eicom, Kyoto, Japan). Each coordinate was made according to an atlas of the mouse brain [26].

For the behavioral analysis, a 0.5 μl/site NeuRet-IL-2Rα-EGFP vector (1.0–5.0 × 10^12^ copies/ml) was injected into the bilateral CL. The rostrocaudal, mediolateral, and dorsoventral coordinates (mm) from the bregma and dura were − 1.5/±0.8/2.9. To investigate whether the DN neurons innervating the CL have collateral axons to other brain regions, NeuRet-Cre vector (3.5 × 10^12^ copies/ml) or PBS was injected into the right CL. Injection was carried out at a constant flow rate of 0.1 μl/min (0.5 μl/site).

For the IT solution injection, recombinant IT, anti-Tac(Fv)-PE38 [27], was diluted to a final concentration of 80 μg/ml in PBS containing 0.1% mouse serum albumin. Then, 1.0 μl/site IT solution or PBS was injected into the bilateral DN. The rostrocaudal, mediolateral, and dorsoventral coordinates (mm) from the bregma and dura were − 5.8/±2.3/2.1 and − 6.2/±2.3/2.1. Injection was carried out at a constant flow rate of 0.2 μl/min (1.0 μl/site).

For the AAV2 vector injection, a AAV2-CAGGS-FLEX-EGFP vector (1.0 × 10^13^ copies/ml) was injected into the left DN. Injection was carried out at a constant flow rate of 0.1 μl/min (0.5 μl/site).

For the ibotenic acid (IBO) injection, IBO (Wako Pure Chemical, Osaka, Japan) was diluted to a final concentration of 0.5 mg/ml in PBS. IBO solution or PBS was injected into the bilateral VA/VL. The rostrocaudal, mediolateral, and dorsoventral coordinates (mm) from the bregma and dura were − 1.0/±1.1/3.36. Injection was carried out at a constant flow rate of 0.1 μl/min (0.1 μl/site).

### Immunohistochemistry

Brain sections were prepared according to our previous report [22]. Immunostaining with the immunoenzyme method and immunofluorescent staining were performed as described previously [22] [28]. Brain sections cut in a cryostat at 30 μm were incubated with primary antibodies for rabbit polyclonal anti-GFP antibody (1:3000; Thermo Fisher Scientific, Waltham, MA) or mouse monoclonal anti-NeuN IgG (1:1000; Millipore Corporate Headquarters, Billerica, MA), and then with secondary antibodies for Cy3-conjugated donkey anti-mouse IgG (1:1000; Jackson ImmunoResearch, West Grove, PA), Alexa488-conjugated goat anti-rabbit IgG (1:400; Invitrogen, Carlsbad, CA), or Biotin-SP-conjugated donkey anti-rabbit IgG (1500; Jackson ImmunoResearch). For immunostaining with the immunoenzyme method, expressions were enhanced using a Vectastain Elite ABC kit (Vector Laboratories, Burlingame, CA) with 3,3′-diaminobenzide tetrahydrochloride/H_2_O_2_ as a chromogen. For fluorescent staining, cell nuclei were counterstained with DAPI. After staining, the sections were scanned using a DM6000B fluorescence microscope (Leica, Wetzlar, Germany) or a LSM510 confocal microscope (Carl Zeiss, Thornwood, NY).

### Behavioral analyses

Male mice were regularly handled and subjected to the following behavioral tests. Each test was performed in the light cycle on separate days. Brightness of the floor was at an intensity of 340 lx. The mice were food-restricted to maintain 85% of free feeding weight before the start of the gait analysis, the single-seed reaching test, the place discrimination test, or the T-maze test to ensure motivation to perform these behavioral tasks. Mice received the bilateral neuronal elimination were used, because neurons in the DN innervate the CL contralaterally and thus bilateral elimination of the DN-CL pathway is required for the analyses of behavioral roles of this pathway.

For the open field test, the apparatus consisted of a gray floor (42 cm × 42 cm) with 30-cm-high gray walls. The floor was divided into a center zone (central 25 cm × 25 cm) and a peripheral zone (other area). Each mouse was placed in the center of the open field, and was allowed to explore freely for 30 min. A video tracking system (Viewer 2; Bioserv, Flemington, NJ) was used to measure the precise trajectory of each mouse. The total distance traveled, the average velocity, and the number of entries into the center zone were measured.

For the gait analysis, the footprint pattern test was used. The apparatus consisting of a transparent floor (100-cm-long and 5-cm-wide) with 15-cm-high transparent walls was used based on a previous study [29]. Mice were acclimated to the food pellets (20 mg; Bioserv) that were used to lead the mice to walk straight along the apparatus. Forelimbs and hindlimbs were coated with blue and red non-toxic paints, respectively, and the mice walked over a sheet of white paper. Four footprints, at least, obtained from individual mice were used for quantification, and the average distance between each footprint was calculated. Footprint pattern was evaluated as described previously [30]. Footprints made at the beginning and end of the run were excluded from the analysis.

A rotarod test was performed as previously described [22]. Briefly, an accelerating rotarod (ENV-575 M; Med Associates, Georgia, FL) was used with a rotation speed that was increased from 4 to 40 rpm over 5 min in each trial. Mice were placed on the rotarod and the time that each mouse was dropped from the rotating rod was measured. The mice were trained with three trials per day with a 30 min intertrial interval for three consecutive days.

For the single-seed reaching test, a reaching box [31] was used with minor modifications as mentioned below. The transparent box (20-cm-tall, 15-cm-deep, and 8.5-cm-wide) had three vertical slits (0.5-cm-high and 13-cm-high) in the center, on the left side, and on the right side of the front wall. A 2.0-cm-tall exterior shelf was placed in front of the slits to place millet seeds (Kurose Pet Foods, Fukuyama, Japan). The experiment was performed as described previously [31] with minor modifications. Briefly, a number of millet seeds were placed in front of the center slit in the acclimation phase, which was performed to familiarize mice with task requirements and also to determine their preferred limbs. Acclimation was considered finished when the mouse attempted to reach the millet seed at least 20 times during 20 min and when the mouse showed > 70% limb preference in two consecutive days. When mice did not reach this criterion within a week, the mice were excluded from the analysis (one mouse in the IT group was excluded from this study). On the day after the completion of the acclimation, motor performance to reach the seed was evaluated. A single seed was presented in front of the slit on the side of the preferred limb. The test consisted of one session of 30 trials with a preferred limb or 20 min (whichever occurred first). Reaching was regarded as ‘success’ when the mouse successfully retrieved the seed. Success rate within 30 reaching attempts was measured.

The two-lever place discrimination test was conducted in an operant chamber (21.6 cm × 17.8 cm × 12.7 cm) equipped with a pellet receptacle in the center of the front panel (ENV-307 W; Med Associates). Two retractable levers (left or right) were mounted on either side of the receptacle. The chamber was enclosed in a sound-attenuating box and illuminated with a house light during the trials. In the acclimation phase (4–8 days in duration), the mice were trained to press either lever, presented at the same time, to obtain the delivery of a 20 mg food pellet (Bioserv). The test consisted of one session of 50 lever presses or 30 min (whichever occurred first). The preferred lever was determined following the delivery of 150 pellets. In the acquisition phase, both levers were presented at the same time, and the mice were reinforced to press the unpreferred lever, which was determined during the acclimation phase. The test consisted of one session of 10 lever presses or 30 min (whichever occurred first) for 7 days. In the reversal learning phase, both levers were presented at the same time, but the “correct” lever in the acquisition phase was switched, and mice were reinforced to press the other lever. The test also consisted of one session of 10 lever presses or 30 min (whichever occurred first) for 7 days. The correct response rate was measured in the acquisition and reversal learning phases.

The Morris water maze test was performed as previously described [32]. Briefly, mice were trained with two trials per day for six (for the acquisition phase) or 4 days (for the flexible learning phase). The position of the platform was moved to the opposite quadrant in the flexible learning phase. The time to reach the platform was measured. The probe tests were conducted 1 day after the final day of the acquisition phase and flexible learning phase. The time spent in each quadrant was measured in a single 60 s trial.

The T-maze test was conducted in a four-arm cross maze, which consists of a central square (8 × 8 cm) equipped with four arms (32 × 8 cm), walls (34 cm height), and a recessed food cup (3 cm in diameter, 1.6 cm in height, placed at the end of each arm). The four-arm cross maze was modified into a T-shape by preventing entrance into one of the arms with a removable wall. Two opposite arms facing north and south were used as choice arms, and the remaining two arms facing east and west were used as start arms. The spatial learning test consisted of three phases including the acclimation phase, the acquisition phase, and the flexible learning phase. In the acclimation phase, mice were placed in the central area of the maze, and were allowed to move freely and get rewards from all arms, which were baited with 50 μl of 30% sucrose solution. In the acquisition phase, one choice arm (correct arm) of the maze was reinforced with 50 μl of 30% sucrose solution, whereas another choice arm (failed arm) was not reinforced. Mice were placed in one of the start arms in a pseudorandom order, and were required to select the correct arm. Mice performed 12 training trials per day (the intertrial interval was at least 10 min) for 10 consecutive days. In the flexible learning phase, the “correct” arm in the acquisition phase was switched, and mice were reinforced to enter the other arm. The test also consisted of 12 training trials per day (the intertrial interval was at least 10 min) for 10 consecutive days. The correct response rate was measured in the acquisition and flexible learning phases.

### Statistical analyses

Two-way repeated analysis of variance (ANOVA) or unpaired Student’s *t*-test was performed. Difference with *p* < 0.05 was considered to be statistically significant. All data are expressed as the mean ± s.e.m.

## Results

### Selective gene expression in thalamic input pathways

**Fig. 1:**
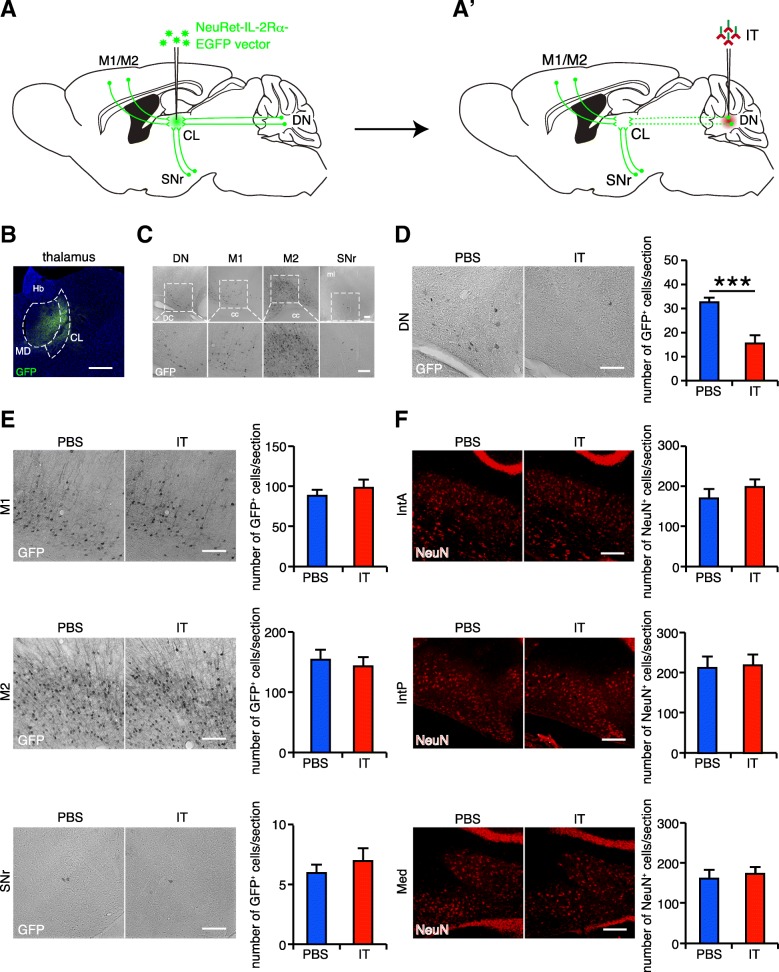
Selective elimination of neurons in the DN innervating the CL. (A and A’) Selective targeting of the cerebellothalamic tracts. Injection of the NeuRet lentiviral vector encoding the human IL-2Rα-EGFP into the CL induced the expression of transgene in various brain regions innervating the CL. Subsequent injection of the IT solution into the DN selectively eliminated the cerebellothalamic tracts. **b** IL-2Rα-EGFP expression around the injection sites. The GFP expression is observed in the CL. **c** IL-2Rα-EGFP expression through retrograde transport. The expression is localized in neurons in the DN, M1, M2, and SNr. cc, corpus callosum; DC, dorsal cochlear nucleus; Hb, habenular; and ml, medial lemniscus. **d** Selective neuronal elimination in the DN. The number of GFP^+^ cells in the DN is decreased by the IT treatment. *n* = 8 mice for the PBS-treated group and *n* = 6 mice for the IT-treated group. **e** Intact number of neurons in several brain regions innervating the CL. The number of GFP^+^ cells in the SNr, M1, or M2 is not affected by the IT treatment. *n* = 4 mice for each group. **f** Intact number of neurons in other deep cerebellar nuclei. The number of NeuN^+^ cells in the IntA, IntP, or Med is not changed by the IT treatment. *n* = 8 mice for the PBS-treated group and *n* = 6 mice for the IT-treated group. ***p < 0.001 (Student’s *t*-test). All data are expressed as the mean ± s.e.m. Scale bars: (B) 400 μm and (C-F) 100 μm

The CL has afferent projections from several brain regions including the DN [18], primary motor cortex (M1), secondary motor cortex (M2) [33], and the substantia nigra pars reticulata (SNr) [34]. To induce the expression of human IL-2Rα in the brain regions innervating the CL, we injected the NeuRet-IL-2Rα-EGFP vector into the CL of mice using stereotaxic surgery (Fig. 1a). After 7 days, we prepared brain sections and stained them with anti-GFP antibody. The GFP-positive signals were observed in the CL (Fig. 1b) and in some input areas including the DN, M1, M2, and SNr (Fig. 1c). These results demonstrate that the IL-2Rα-EGFP expression in the input regions generated via retrograde gene transfer of the vector.

We also detected the weak expression of GFP in the mediodorsal nucleus (MD) of the thalamus in several mice (Fig. 1b). Neurons in the DN are reported to innervate the MD in monkeys [35], but this projection is not observed in mice [19] or rats [36]. Thus, the expression of IL-2Rα-EGFP in the MD does not influence any cerebellothalamic elimination in this study.

### Selective elimination of the cerebellothalamic tracts

IT recognizes the neurons harboring IL-2Rα and induces selective removal of the targeted neuronal tracts [20]. To eliminate the cerebellothalamic tracts, mice received a bilateral injection of the NeuRet-IL-2Rα-EGFP vector, and then were bilaterally injected with PBS or the IT solution into the DN (Fig. 1a‘). After the PBS/IT treatment, the brains were processed, and sections were prepared for histological analyses. We quantified the number of GFP-positive cells in various regions that innervate the CL. The number of GFP-positive cells was significantly decreased in the DN of the IT-treated mice compared to that in the DN of the PBS-treated mice (47.5% of the PBS-treated controls, *t*_12_ = 4.928, *p* < 0.001, Fig. 1d). No difference in the number of GFP-positive cells was detected in the M1, M2, or SNr (*t*_6_ = 0.800, *p* = 0.454 for M1; *t*_6_ = 0.468, *p* = 0.656 for M2; and *t*_6_ = 0.722, *p* = 0.497 for SNr, Fig. 1e), showing that the corticothalamic and nigrothalamic tracts were intact. We also checked the effect of treatment on other deep cerebellar nuclei, such as anterior interpositus nucleus (IntA), posterior interpositus nucleus (IntP), and medial nucleus (Med). We evaluated the number of NeuN-positive cells in these nuclei because the expression of GFP in these regions were rarely observed. The number of NeuN-positive cells in these nuclei was not altered by the IT treatment (*t*_12_ = 0.940, *p* = 0.366 for IntA; *t*_12_ = 0.181, *p* = 0.859 for IntP; and *t*_12_ = 0.397, *p* = 0.698 for Med, Fig. 1f). These results demonstrate that the IT treatment caused a selective elimination of the DN-derived cerebellothalamic tracts in the vector-injected mice.

### Cerebellothalamic elimination does not change overground locomotion

**Fig. 2:**
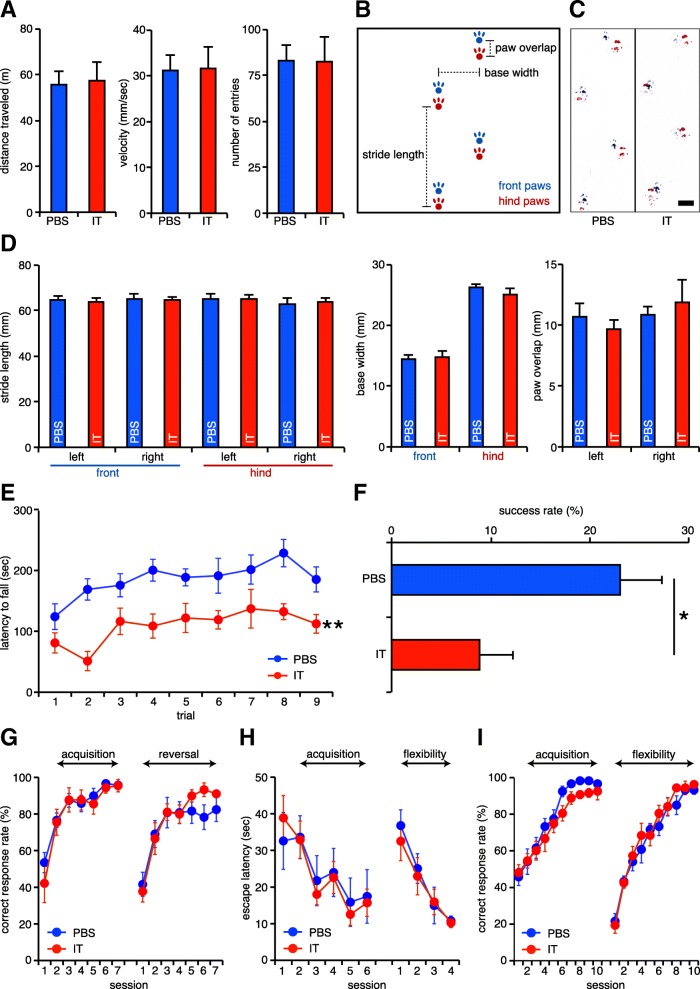
Impaired motor skills in mice lacking the cerebellothalamic tracts. **a** Intact spontaneous overground locomotion. Distance traveled, velocity, or the number of entries into the center zone of the open field are not affected by the IT treatment. *n* = 8 mice for the PBS-treated group and *n* = 6 mice for the IT-treated group. **b** Parameters measured in the gait analysis. **c** Representative walking footprint patterns of each mouse. No obvious difference between the groups is observed. **d** Quantitative analyses of the footprints. Stride length of each limb, base width of front limbs or hind limbs, or paw overlap of left limbs or right limbs are not altered by the IT treatment. *n* = 8 mice for the PBS-treated group and *n* = 6 mice for the IT-treated group. **e** Impaired motor coordination in mice lacking the cerebellothalamic tracts. Mice treated with the IT solution show shorter latency until fall in the rotarod test. *n* = 12 mice for the PBS-treated group and *n* = 9 mice for the IT-treated group. **p < 0.01 (two-way ANOVA). **f** Impaired forelimb reaching in mice lacking the cerebellothalamic tracts. The success rate in the single-seed reaching test is decreased in mice treated with the IT solution. *n* = 8 mice for the PBS-treated group and *n* = 6 mice for the IT-treated group. *p < 0.05 (Student’s *t*-test) **g** Intact correct response rate in the place discrimination test. The correct response rate is not affected in either learning phase by the IT treatment. *n* = 12 mice for the PBS-treated group and *n* = 9 mice for the IT-treated group. **h** Similar escape latency in the Morris water maze test. The latency to find the platform is not affected in either learning phase by the IT treatment. *n* = 8 mice for each group. **i** Normal correct response rate in the T-maze test. The correct response rate is not affected in either learning phase by the IT treatment. *n* = 10 mice for the PBS-treated group and *n* = 9 mice for the IT-treated group. All data are expressed as the mean ± s.e.m. Scale bar: (**c**) 10 mm

We evaluated the impact of the cerebellothalamic elimination on some behavioral tasks that are related to the cerebellar functions. We first examined locomotor activity of the PBS/IT-treated mice in the open field test. The mice showed similar values in the distance traveled and velocity between the PBS- and IT- treated groups (*t*_12_ = 0.097, *p* = 0.925, Fig. 2a). In addition, the IT-treated mice spent similar time in the center zone compared to the PBS-treated mice (*t*_12_ = 0.051, *p* = 0.960, Fig. 2a), suggesting normal anxiety-like behavior of the mice lacking the cerebellothalamic tracts. These results show that elimination of the cerebellothalamic tracts does not alter spontaneous locomotion.

Ataxia is one of the most known symptoms of cerebellar dysfunction [37]. Here we investigated gait of the PBS/IT-treated mice by using footprint analysis (Fig. 2b). Typical gait patterns of the two mouse groups are shown in Fig. 2c. We found no difference in the values that characterize the gait patterns, including stride length, base width, and paw overlap between the two groups (*t*_12_ = 0.234, *p* = 0.819, stride length of the left front paw; *t*_12_ = 0.133, *p* = 0.896, stride length of the right front paw; *t*_12_ = 0.133, *p* = 0.896, stride length of the left hind paw; *t*_12_ = 0.111, *p* = 0.913, stride length of the right hind paw; *t*_12_ = 0.257, *p* = 0.801, stride length of the right hind paw; *t*_12_ = 0.318, *p* = 0.756, base width of the front paw; *t*_12_ = 1.061, *p* = 0.309, base width of the hind paw; *t*_12_ = 0.666, *p* = 0.518, overlap of the left paw; *t*_12_ = 0.562, *p* = 0.584, overlap of the right paw, Fig. 2d). Thus, the cerebellothalamic elimination does not cause cerebellar ataxia.

### Cerebellothalamic elimination impairs motor skills

The cerebellum is involved in several types of motor skill control, including motor coordination [38, 39] and forelimb reaching [5]. Here we investigated such complex motor skills using mice that lacked the cerebellothalamic tracts projecting to the CL.

To evaluate motor coordination, the PBS- and IT-treated mouse groups were subjected to the rotarod test. The latency to fall from the rotating rod in the IT-treated mice was significantly shorter than that of the PBS-treated mice, but the latency increased along with the trial number similarly between the PBS- and IT-treated mice (group effect, *F*_(1,19)_ = 13.327, *p* = 0.002; trial effect, *F*_(8,152)_ = 5.044, *p* < 0.001; group x trial interaction, *F*_(8,152)_ = 0.980, *p* = 0.454, two-way ANOVA, trial as repeated measure) (Fig. 2e). The latency to fall of the PBS- and IT-treated mice reached the plateau within several trials (three to four trials), and the latency to fall of the IT-treated mice did not reach that of the PBS-treated mice even during nine trials. Thus, elimination of the cerebellothalamic tracts impairs motor coordination with no significant influence on motor skill learning.

To further test more refined motor skill behavior, the two mouse groups were conducted for the single-seed reaching test, which evaluates the coordinated forelimb movement. In this test, mice were trained to grasp and retrieve a food pellet through a slit using the front paw. The success rate of reaching was decreased in the IT-treated mice compared to that in the PBS-treated mice (*t*_12_ = 2.473, *p* = 0.029, Fig. 2f). These data support that the cerebellothalamic tracts are required for the control of motor skills.

### Cerebellothalamic elimination does not impair place discrimination or its reversal learning

The cerebellum is not only involved in the motor skill control but also in discrimination learning [6]. We have recently shown that neurons in the CL innervating the DS are involved in the reversal learning of place discrimination with intact acquisition learning [24]. In the current study, we evaluated the role of the cerebellothalamic tracts in the place discrimination test using the two-lever choice task dependent on place cues [24]. In the acquisition phase, the correct response rate was not affected between the PBS- and IT-treated mice (group effect, *F*_(1,19)_ = 0.312, *p* = 0.583; session effect, *F*_(6,114)_ = 30.964, *p* < 0.001; group x session interaction, *F*_(6,114)_ = 0.545, *p* = 0.773, two-way ANOVA, session as repeated measure) (Fig. 2g). The correct response rate in the reversal learning was also not affected between these two groups (group effect, *F*_(1,19)_ = 0.273, *p* = 0.608; session effect, *F*_(6,114)_ = 26.751, *p* < 0.001; group x session interaction, *F*_(6,114)_ = 1.163, *p* = 0.331, two-way ANOVA, session as repeated measure) (Fig. 2g). These data demonstrate that the cerebellothalamic tracts are not involved in place discrimination learning.

### Pathway elimination does not disturb spatial recognition or its flexibility

Several studies have reported that the cerebellum is involved in spatial learning during the Morris water maze test both in the acquisition and flexible learning phases [7, 8]. We therefore examined the involvement of the cerebellothalamic tracts in spatial learning by using the Morris water maze test. In the acquisition phase, the escape latency was not changed between the PBS- and IT-treated mice (group effect, *F*_(1,14)_ = 0.061, *p* = 0.809; session effect, *F*_(5,70)_ = 4.657, *p* = 0.001; group x session interaction, *F*_(5,70)_ = 0.197, *p* = 0.963, two-way ANOVA, session as repeated measure) (Fig. 2h). During the probe trial following the acquisition phase, the PBS-treated mice spent 24.7 ± 3.2 s in the correct quadrant, while the IT-treated mice spent 21.3 ± 3.4 s (*t*_14_ = 0.732, *p* = 0.476) in the 60 s trial. In the flexible learning phase, the platform was relocated in the opposite quadrant. The escape latency also did not differ between the two mouse groups (group effect, *F*_(1,14)_ = 0.262, *p* = 0.617; session effect, *F*_(3,42)_ = 14.064, *p* < 0.001; group x session interaction, *F*_(3,42)_ = 0.157, *p* = 0.925, two-way ANOVA, session as repeated measure) (Fig. 2h). During the probe trial following the flexible learning phase, the PBS-treated mice spent 21.2 ± 1.9 s in the correct quadrant, while the IT-treated mice spent 19.5 ± 3.8 s (*t*_14_ = 0.386, *p* = 0.706) in the 60 s trial.

We further tested the spatial learning ability of the mice using the T-maze test. In the acquisition phase, the correct response rate was not affected between the PBS- and the IT-treated mice (group effect, *F*_(1,17)_ = 2.602, *p* = 0.125; session effect, *F*_(9,153)_ = 43.054, *p* < 0.001; group x session interaction, *F*_(9,153)_ = 0.608, *p* = 0.789, two-way ANOVA, session as repeated measure) (Fig. 2i). In the flexible learning phase, the correct arm was relocated to the opposite arm. The correct response rate did not differ between the two groups (group effect, *F*_(1,17)_ = 0.481, *p* = 0.497; session effect, *F*_(9,153)_ = 80.410, *p* < 0.001; group x session interaction, *F*_(9,153)_ = 0.655, *p* = 0.749, two-way ANOVA, session as repeated measure) (Fig. 2i). These results consistently showed that the functions of spatial learning and its flexibility were normally preserved in the mice that underwent cerebellothalamic elimination.

### Axonal arborization of the DN neurons innervating the CL

**Fig. 3:**
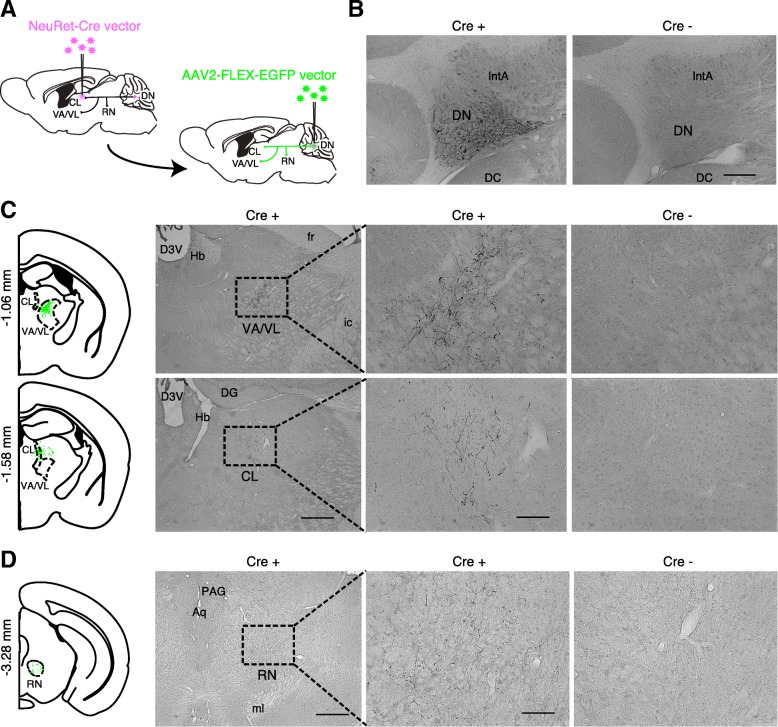
Axonal arborization of the DN neurons innervating the CL to the VA/VL. **a** Selective targeting of axonal arborization of the DN neurons innervating the CL. Injection of the NeuRet lentiviral vector encoding Cre into the CL induced the expression of Cre in various brain regions innervating the CL, including the DN. Subsequent injection of the AAV2-FLEX-EGFP into the DN selectively labels axonal terminals branched from the DN-CL tracts. **b** GFP expression in the DN. The GFP expression is selectively observed in the DN of mice injected with the NeuRet-Cre vector. **c** GFP expression in the VA/VL and the CL. The GFP expression is observed in the rostral VA/VL and the CL of mice injected with the NeuRet-Cre vector. **d** GFP expression in the RN. Only a small number of GFP expression is observed in the RN of mice injected with the vector. Scale bars: (**b**) 200 μm, (**c**) 400 μm for lower magnification and 100 μm for higher magnification, and (**d**) 400 μm. Abbreviations: Aq, aquedact; D3V, dorsal third ventricle; DC, dorsal cochlear nucleus; DG, dentate gyrus; fr, fasciculus retroflexus; Hb, habenular; ic, internal capsule; ml, medial lemniscus; PAG, periaqueductal gray

In the present study, we selectively eliminated the DN neurons innervating the CL using IT targeting technology. However, we need to define whether the DN neurons projecting to the CL have collateral axons to other brain regions. DN neurons are known to have massive projections to the VA/VL and the red nucleus (RN) [12]. To investigate whether the DN neurons innervating the CL have collateral axons to the VA/VL and the RN, the NeuRet-Cre vector was unilaterally injected into the CL, and then the AAV2-FLEX-EGFP vector was injected into the DN in the contralateral side (Fig. 3a). GFP expression was detected in DN neurons only in the part of the brain where the vector encoding Cre gene was injected (Fig. 3b). A number of GFP-positive axonal terminals were visualized in the CL and the rostral area of the VA/VL in the contralateral side of the DN (Fig. 3c). However, the number of the positive terminals was very minor in the RN (Fig. 3d). Thus, the DN neurons projecting to the CL have axonal collaterals innervating the rostral VA/VL area.

**Fig. 4:**
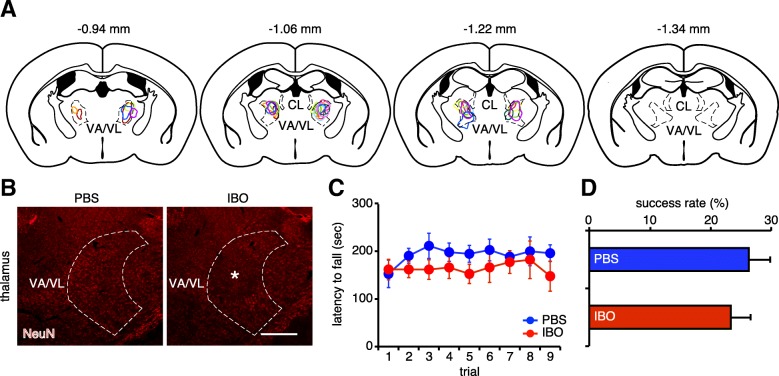
No detectable behavioral contribution by the rostral VA/VL lesion. **a** Schematic illustration showing lesion area in an individual mouse. **b** Representative lesion area by IBO treatment. Center of the lesion area is shown by asterisk. **c** Intact motor coordination in mice with VA/VL lesion. The latency to fall in the rotarod test is not influenced by the IBO treatment. *n* = 8 mice for the PBS-treated group and *n* = 10 mice for the IBO-treated group. **d** Intact forelimb reaching in mice with VA/VL lesion. The success rate in the single-seed reaching test is not affected by the IBO treatment. *n* = 8 mice for the PBS-treated group and *n* = 10 mice for the IBO-treated group. All data are expressed as the mean ± s.e.m. Scale bar: (**b**) 200 μm

Deletion of axonal collaterals of the DN neurons innervating the rostral VA/VL may be implicated in the aforementioned behavioral changes in the IT-treated mice. To evaluate behavioral contributions of the rostral VA/VL, mice received a bilateral injection of the IBO solution into the corresponding region. The range of IBO lesions was detected between − 0.94 mm to − 1.22 mm from the bregma, although the VA/VL was observed between − 0.94 mm to − 1.70 mm from the bregma (Fig. 4a and b). We then conducted the analysis of motor skill control. In the rotarod test, there was no statistical difference in the latency to fall from the rotating rod between the PBS- and IBO-treated mice (group effect, *F*_(1,16)_ = 1.545, *p* = 0.232; trial effect, *F*_(8,128)_ = 0.504, *p* = 0.851; group x trial interaction, *F*_(8,128)_ = 0.454, *p* = 0.886, two-way ANOVA, trial as repeated measure), although the IBO-treated mice did not seem to extend the latency even through nine trials (Fig. 4c). In the reaching test, the success rate was indistinguishable between the PBS- and IBO-treated mice (*t*_16_ = 0.623, *p* = 0.542, Fig. 4d). Thus, disruption of the rostral VA/VL had no detectable behavioral contribution in both tests. The behavioral deficits observed by the eliminated DN neurons innervating the CL did not appear to be confounded by the deletion of axonal arborization to the rostral VA/VL.

## Discussion

In the present study, we analyzed the behavioral contributions of the cerebellothalamic tracts originating from the DN and projecting to the CL using a combination of IT targeting technology of specific neuronal pathways and pharmacological neuronal ablation. We found that the DN-CL tracts are involved in motor skills but not in spatial recognition functions. Our findings suggest that the CL mediates the cerebellar motor information, although the DN-CL tracts are relatively minor compared to the DN-VA/VL tracts [36].

The CL is involved in various aspects of the motor function. A previous report shows that electrolytic lesions in the CL of rats reduce latency during the late learning phase but not during the early phase in the rotarod test [40]. This inconsistent result, which compared to ours, might be derived from the difference in the experimental conditions. The authors performed the rotarod test with a fixed speed of 30 rpm; however, we performed the test with an accelerating speed of 4 to 40 rpm over 5 min. This experimental difference indicates that their behavioral task may be more difficult to perform than our task. In fact, the latency to fall in the first trial of sham mice in their condition was shorter than that of the PBS-treated mice in the current study. In addition, the latency to fall did not plateau even after 10 trials in their condition; however, it reached the plateau within four trials in our condition. It is therefore possible that the CL may also be involved in the learning of motor skills, although the function depends on the difficulty of the task.

The cerebellar motor information is supposed to be further integrated in other brain regions. The main target regions assumed are the M1/M2, which are involved in motor control and have reciprocal projections with the CL [13, 33]. Although neurons in the CL also innervate the DS [17–19], we have shown that the elimination of the CL-DS tracts using the IT targeting technology did not impair motor performance in the rotarod test [24]. Thus, the cerebellar motor information, through the CL, may be processed in the M1/M2 areas.

In this study, we identified the axonal collaterals of the DN-CL tracts to the rostral VA/VL, which are different from the ordinary cerebellothalamic tracts. The rostromedial portion of the VA/VL receives inhibitory afferents from the basal ganglia, and the caudolateral portion of the VA/VL receives excitatory afferents from the cerebellar nuclei [41]; thus, the rostral VA/VL is likely involved in the processing of behavioral information from the basal ganglia, although no detectable motor deficit was observed in the present study. In the rotarod test, we found that there was no significant impact in the group effect between the PBS- and IBO-treated mice. However, the IBO-treated mice did not seem to improve their motor performance even through nine trials. This is consistent with a previous report showing that the electrolytic lesions in whole VA/VL and the ventromedial nucleus of the thalamus impaired motor learning, not basal motor performance, in the rotarod test [42]. Local neuronal elimination in the rostral VA/VL does not induce apparent motor deficits, but more broad neuronal elimination in the VA/VL may cause more apparent dysfunction in motor skill learning.

Our results reveal that the DN-CL tracts have behavioral contributions in motor skills but not in spatial learning. A previous report showed that electrolytic lesions in the DN prolonged escape latency, but reached similar level compared to the sham group after several trials in the Morris water maze test with intact performance in the probe trials [7]. These results suggest that the DN is involved in the spatial learning speed, but not in the memory retention. On the contrary, our data show that the DN-CL tracts do not affect the learning speed in the Morris water maze test or in the T-maze test. Thus, other efferent projections from the DN, especially to the VA/VL, may have roles in processing cerebellar spatial information [42].

In the present study, we identified a key role of cerebellothalamic pathways arising from the DN and projecting to the CL in the control of coordinated motor skill functions. The detailed mechanism that the DN-CL tracts regulate such motor skills cooperating with the motor cortex and basal ganglia circuits should be addressed in future studies.

## Abbreviations

AAV: Adeno-associated virus
ANOVA: Analysis of variance
CL: Central lateral nucleus
DN: Dentate nucleus
DS: Dorsal striatum
EGFP: Enhanced green fluorescent protein
IBO: Ibotenic acid
IL-2Rα: Interleukin-2 receptor α-subunit
IntA: Anterior interpositus nucleus
IntP: Posterior interpositus nucleus
IT: Immunotoxin
M1: Primary motor cortex
M2: Secondary motor cortex
MD: Mediodorsal nucleus
Med: Medial nucleus
NeuRet: Neuron-specific retrograde gene transfer
RN: Red nucleus
SNr: Substantia nigra pars reticulata
VA: Ventroanterior thalamic nucleus
VL: Ventrolateral thalamic nucleus
